# Evolving beta-lactamase epidemiology in *Enterobacteriaceae* from Italian nationwide surveillance, October 2013: KPC-carbapenemase spreading among outpatients

**DOI:** 10.2807/1560-7917.ES.2017.22.31.30583

**Published:** 2017-08-03

**Authors:** Tommaso Giani, Alberto Antonelli, Mariasofia Caltagirone, Carola Mauri, Jessica Nicchi, Fabio Arena, Elisabetta Nucleo, Silvia Bracco, Annalisa Pantosti, The AMCLI-CoSA survey participants, Francesco Luzzaro, Laura Pagani, Gian Maria Rossolini

**Affiliations:** 1 Department of Medical Biotechnologies, University of Siena, Siena, Italy; 2 These authors contributed equally to this work; 3 Department of Experimental and Clinical Medicine, University of Florence, Florence, Italy; 4 Department of Clinical, Surgical, Diagnostic, and Paediatric Sciences, Section of Microbiology, University of Pavia, Pavia, Italy; 5 Microbiology and Virology Unit, Department of Laboratory Medicine, A. Manzoni Hospital, Lecco, Italy; 6 Department of Infectious, Parasitic and Immune-Mediated Diseases, Italian National Health Institute, Rome, Italy; 7 The AMCLI-CoSA survey participants are listed at the end of the article; 8 Clinical Microbiology, Virology and Serology Unit, Florence Careggi University Hospital, Florence, Italy

**Keywords:** ESBL, carbapenemase, epidemiology, outpatients

## Abstract

Extended-spectrum beta-lactamases (ESBLs), AmpC-type beta-lactamases (ACBLs) and carbapenemases are among the most important resistance mechanisms in *Enterobacteriaceae*. This study investigated the presence of these resistance mechanisms in consecutive non-replicate isolates of *Escherichia coli* (n = 2,352), *Klebsiella pneumoniae* (n = 697), and *Proteus mirabilis* (n = 275) from an Italian nationwide cross-sectional survey carried out in October 2013. Overall, 15.3% of isolates were non-susceptible to extended-spectrum cephalosporins but susceptible to carbapenems (ESCR-carbaS), while 4.3% were also non-susceptible to carbapenems (ESCR-carbaR). ESCR-carbaS isolates were contributed by all three species, with higher proportions among isolates from inpatients (20.3%) but remarkable proportions also among those from outpatients (11.1%). Most ESCR-carbaS isolates were ESBL-positive (90.5%), and most of them were contributed by *E. coli* carrying *bla*_CTX-M_ group 1 genes. Acquired ACBLs were less common and mostly detected in *P. mirabilis*. ESCR-carbaR isolates were mostly contributed by *K. pneumoniae* (25.1% and 7.7% among *K. pneumoniae* isolates from inpatients and outpatients, respectively), with *bla*_KPC_ as the most common carbapenemase gene. Results showed an increasing trend for both ESBL and carbapenemase producers in comparison with previous Italian surveys, also among outpatients.

## Introduction

*Enterobacteriaceae* are the most common cause of healthcare associated infections, and beta-lactams are among the most used antibiotics in clinical practice for treatment of these infections [1,2]. During the last decades, *Enterobacteriaceae* with decreased susceptibility to beta-lactams have been increasingly reported worldwide, causing major problems [3-8].

The most important resistance mechanism to beta-lactams in *Enterobacteriaceae* is the production of beta-lactamases, and the most challenging enzymes of this family are the extended-spectrum beta-lactamases (ESBLs), the AmpC-type beta-lactamases (ACBLs), and the carbapenemases [9-12]. ESBLs are able to hydrolyse a wide range of beta-lactams, including penicillins, narrow- and extended-spectrum cephalosporins and monobactams, but not cephamycins and carbapenems. ESBLs are usually inhibited by conventional beta-lactamase inhibitors. CTX-M-type ESBLs, which emerged in the late 1980s, and demonstrated a high ability to disseminate in clinical settings, rapidly reaching a pandemic diffusion. Nowadays they are the most prevalent plasmid-encoded ESBLs overall [13,14]. Acquired ACBLs can confer resistance to penicillins and to most cephalosporins (including cephamycins) but are poorly or not active against monobactams and the zwitterionic oxyimino-cephalosporins, such as cefepime and cefpirome, and the carbapenems. ACBLs are generally less prevalent than ESBLs in *Enterobacteriaceae* but are nonetheless important for their contribution to beta-lactam resistance, which can be extended also to carbapenems when ACBLs are overproduced in combination with an impermeability defect [10,15,16]. Carbapenemase production is the leading resistance mechanism to carbapenems in *Enterobacteriaceae*. Acquired carbapenemases of the KPC-, VIM-, NDM- and OXA-48-types are the most prevalent, although with a notable geographical variability [6,17-20].

In Italy, the most recent data from the European Antimicrobial Resistance Surveillance Network (EARS-Net) reported very high proportions of resistance to extended-spectrum cephalosporins among *Escherichia coli* (30.1%) and *Klebsiella pneumoniae* (55.9%), and of resistance to carbapenems among *K. pneumoniae* (33.5%) [21]. However, the EARS-NET data neither cover other enterobacterial species nor describe the resistance mechanisms.

This survey, promoted by the Committee for Study of Antibiotics (CoSA) of the Italian Society of Clinical Microbiologists (AMCLI), was carried out to provide an updated picture of the molecular epidemiology of ESBL- and carbapenemase-producing *Enterobacteriaceae* circulating in Italy and to investigate, for the first time, the presence of ACBL producers on a nationwide scale.

## Methods

### Study design

**Figure 1:**
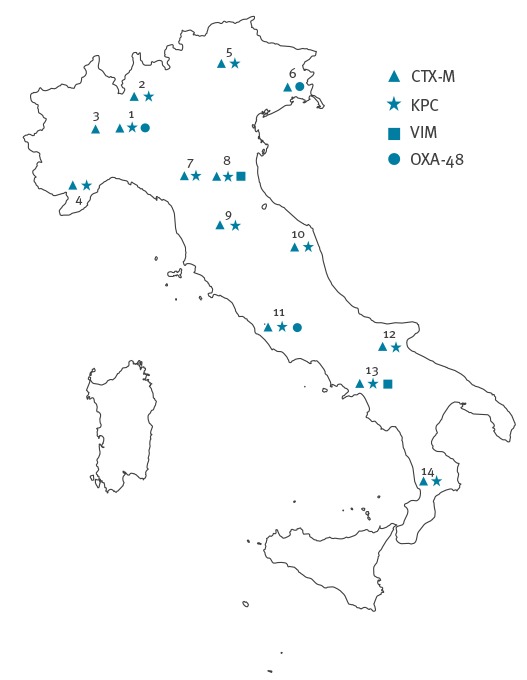
Distribution of the centers participating in the survey, Italy, October 2013 (n=14)

Fourteen clinical microbiology laboratories from 13 Italian cities participated in the study (Figure 1).

For each centre the presence of CTX-M extended-spectrum beta-lactamase and carbapenemase genes is also indicated.

Centers listed as follows: 1-Milano; 2-Lecco; 3-Novara; 4-San Remo; 5-Bolzano; 6-Udine; 7-Modena Bg; 8-Modena Pc; 9-Firenze; 10-Ancona; 11-Roma; 12-San Giovanni Rotondo; 13-Avellino; 14-Cosenza.

The laboratories were selected so as to provide a countrywide coverage by large laboratories associated with hospitals, representative of most Italian Regions. Twelve of the 14 laboratories had also been involved in the first Italian survey on carbapenemase-producing *Enterobacteriaceae*, carried out in 2011 [22]. The survey period was from 1 to 15 October 2013.

The laboratories consecutively collected all non-replicate (only first isolate from a patient included) clinical isolates of *E. coli*, *K. pneumoniae* and *Proteus mirabilis,* from any site of infection, showing a minimum inhibitory concentration (MIC) > 1 mg/L for extended-spectrum cephalosporins (ESC) (cefotaxime and/or ceftriaxone and/or ceftazidime and/or cefepime), and/or for ertapenem. Participating laboratories determined MICs of ESC and ertapenem by the automated systems routinely used in the respective laboratory: either Vitek 2 (bioMérieux, Marcy l’Etoile, France) or BD Phoenix (Becton, Dickinson and Co., New Jersey, United States (US)). The collected isolates were sent to reference laboratories for confirmation of species identification and characterisation of the resistance mechanisms.

For each isolate, information on the type of clinical specimen and of patient (inpatient or outpatient) were provided. Isolates from patients from nursing homes or other long-term care facilities, and isolates from surveillance specimens, were excluded. Each laboratory also provided information on the total number of non-replicate clinical isolates of *Enterobacteriaceae* of the same species observed during the collection period from inpatients and outpatients.

### Bacterial identification, antimicrobial susceptibility testing and phenotypic characterisation of resistance mechanisms

At the reference laboratories, identification of collected isolates was confirmed by MALDI-TOF mass spectrometry (Vitek MS, bioMérieux), and susceptibility to ESC (cefotaxime, ceftazidime, cefepime), carbapenems (ertapenem, imipenem, and meropenem), aztreonam, aminoglycosides (amikacin and gentamicin), ciprofloxacin, trimethoprim/sulfamethoxazole was determined by disc diffusion on Mueller-Hinton agar (for *P. mirabilis* imipenem was not tested) according to the European Committee on Antimicrobial Susceptibility Testing (EUCAST) methodology [23]. MICs of tigecycline and colistin for carbapenemase producers were determined using reference broth microdilution [24] with custom lyophilised plates (TREK Diagnostic Systems, Cleveland, Ohio, US). All results were interpreted according to the EUCAST breakpoints [25]. ESBL production was investigated using the double disk method testing the synergistic activity between amoxicillin-clavulanate and cefotaxime, ceftazidime, cefepime, and aztreonam [26].

### Molecular characterisation of resistance genes

The presence of carbapenemase genes was investigated by multiplex real time-PCR (mRT-PCR) targeting *bla*_KPC-type,_*bla*_VIM-type_, *bla*_OXA-48-type_ and *bla*_NDM-type_ genes, using primers and conditions reported in Table 1. The presence of *bla*_CTX-M_ ESBL genes was investigated by a mRT-PCR, able to distinguish among different *bla*_CTX-M-type_ variants of groups 1, 2, 8/25 and 9, using primers and conditions reported in Table 1. The presence of ACBL genes was investigated by a multiplex PCR targeting genes encoding ACBLs, as described previously [27]. For *E. coli*, specific primers designed to distinguish between acquired and chromosomal ACBL genes were used [27]. The presence of *mcr-1*-like genes was investigated by a novel real time-PCR (Table 1).

**Table 1:** Sequence of primers and probes used in nationwide surveillance survey of the molecular epidemiology of ESBL- and carbapenemase-producing *Enterobacteriaceae*, Italy, October 2013

Target	Primer name	Sequence (5’-3’)^a^	Reference	Positive control
	OXA-48-like-rt-F	GTAGCAAAGGAATGGCAAGAAA	[44]	*Escherichia coli* ECBZ-1 (*bla*_OXA-48_) [45]
*bla*_OXA-48-like_ genes	OXA-48-like-rt-R	GATGCGGGTAAAAATGCTTG		
	OXA-48-like-rt-P	HEX-CTCTGGAATGAGAATAAGCAGCAAGG-BHQ-1		
*bla*_KPC_ genes	KPC-rt-F	GATACCACGTTCCGTCTGG	[46]	*Klebsiella pneumoniae* FIPP-1 (*bla*_KPC-3_) [47]
	KPC-rt-R	GCAGGTTCCGGTTTTGTCTC		
	KPC-rt-P	FAM-AGCGGCAGCAGTTTGTTGATTG-BHQ-1		
*bla*_VIM_ genes	VIM-rt-F	TGGTCTCATTGTCCGTGATG	[48]	*K. pneumoniae* VA-416/02 (*bla*_VIM-4_) [49]
	VIM-rt-R	CATGAAAGTGCGTGGAGA		
	VIM-rt-P	ROX-AAGCAAATTGGACTTCCCGTAACGC-BHQ-2		
*bla*_NDM_ genes	blaNDM1_F	CGCAACACAGCCTGACTTT	[50]	*E. coli* CVB-1 (*bla*_NDM-1_) [51]
	blaNDM1_R	TCGATCCCAACGGTGATATT		
	blaNDM1_P	CY5-CAACTTTGGCCCGCTCAAGGTATTT-BHQ-3		
*bla*_CTX-M_ group 1 genes	CTX-M-group-1_FW	AAAAATCACTGCGCCAGTTC	[52]	*E. coli* V460a (*bla*_CTX-M-15_), in-house control
	CTX-M-group-1_REV	AGCTTATTCATCGCCACGTT		
	CTX-M-group1-P	HEX-TGGCGACGGCAACCGTCACGCTGTT-BHQ-1	This study	
*bla*_CTX-M_ group 2 genes	CTX-M-group-2_FW	CGACGCTACCCCTGCTATT	[52]	*E. coli* C277a (*bla*_CTX-M-2_), in-house control
	CTX-M-group-2_REV	CCAGCGTCAGATTTTTCAGG		
	CTX-M-group2-P	FAM-TATTGAGCGTGGGCTCGGTTCTGTCCAG-BHQ-1	This study	
*bla*_CTX-M_ group 8/25 genes	CTX-M-group-8/25_ FW	CGATACCACCACGCCATTAG	This study	*E. coli* M26a (*bla*_CTX-M-8_), in-house control
	CTX-M-group-8/25_REV	AACCCACGATGTGGGTAGC	[52]	
	CTX-M-group8/25-P	CY5-CCTGAATGCTGGCAGCGCCGGTG-BHQ-3	This study	
*bla*_CTX_ *_-_*_M_ group 9 genes	CTX-M-group-9_FW	CAAAGAGAGTGCAACGGATG	[52]	*E. coli* V404a (*bla*_CTX-M-14_) [53]
	CTX-M-group-9_REV	ATTGGAAAGCGTTCATCACC		
	CTX-M-group9-P	ROX-CGTGCATTCCGCTGCTGCTGGGCA-BHQ-2	This study	
All *bla*_CTX-M_ genes	U-CTX-M- FW	ATYRAYACMGCVGATAAYWCGCA	This study	*E. coli* V460a (*bla*_CTX-M-15_), *E. coli* C277a (*bla*_CTX-M-2_), *E. coli* M26a (*bla*_CTX-M-8_), *E. coli* V404a (*bla*_CTX-M-14_)
	U-CTX-M- REV	CSGCAATSGGRTTRTAGTTAAC	This study	
	U-CTX-M-P	CY5.5-ATGTGCAGYACCAGTAARGTKATGGC-BHQ-3	This study	
PhHV (internal control)	PhHV-267s	GGGCGAATCACAGATTGAATC	[54]	PhHV DNA cloned in pGEM-T-easy *E. coli* DH5α
	PhHV-337as	GCGGTTCCAAACGTACCAA		
	PhHV-305tq	Cy5.5 -TTTTATGTGTCCGCCACCATCTGGATC-BHQ-3		
*mcr-1-*like genes	Mcr-1-rt-fwd	ATCAGCCAAACCTATCCCATC	This study	*E. coli* FI-4531 (*mcr-1*) [55], *K. pneumoniae* KP-6884 (*mcr-1.2*) [33]
	Mcr-1-rt-rev	ACACAGGCTTTAGCACATAGC		
	Mcr-1-rt-p	Cy5-GACAATCTCGGCTTTGTGCTGACGATC-BHQ-3		

ESBL: extended-spectrum beta-lactamases.

^a^ The amplification programme consisted of 35 two-step cycles of 15s at 95 °C and 60s at 60 °C.

### Spectrophotometric assay for carbapenemase activity

In carbapenem non-susceptible isolates that tested negative for known carbapenemase genes, carbapenemase production was further investigated by measuring the imipenem-hydrolysing specific activity in bacterial crude extracts as described previously [28], using a Cary 100 UV-Vis spectrophotometer (Varian, Walnut Creek, California, US).

### Statistical analysis

Statistical evaluation of differences between resistance rates observed in this study and previous surveillance studies was carried out with the chi-squared test with Yates correction, using the Stata Statistical Software (release 13, College Station, Texas, US). Since the surveillance studies were overall similar by design but there were a few sampling differences, which could act as confounding factors, the tests were treated rather as exploratory analyses, which may indicate trends.

## Results

### Prevalence of *Enterobacteriaceae* with resistance mechanisms to extended-spectrum cephalosporins and/or carbapenems

During the study period, a total of 3,324 consecutive non-replicate clinical isolates of *Enterobacteriaceae* of the target species were isolated in the 14 laboratories participating in the survey, including 2,352 (70.7%) *E. coli*, 697 (21.0%) *K. pneumoniae,* and 275 (8.3%) *P. mirabilis*. Of these, 1,509 isolates (45.4%) were from inpatients and 1,815 (54.6%) from outpatients (Table 2).

**Table 2:** Proportions of ESCR-carbaS and ESCR-carbaR of *Enterobacteriaceae* by species included and origin of isolate, nationwide surveillance survey, Italy, October 2013 (n=3,324 isolates)

Species	Isolates from inpatients							Isolates from outpatients							All isolates						
**Total**	**ESCR**	**%**	**ESCR‑carbaS**	**%**	**ESCR‑carbaR**	**%**	**Total**	**ESCR**	**%**	**ESCR‑carbaS**	**%**	**ESCR‑carbaR**	**%**	**Total**	**ESCR**	**%**	**ESCR‑carbaS**	**%**	**ESCR‑carbaR**	**%**
*Escherichia coli*	920	230	25.0	219	23.8	11	1.2	1,432	162	11.3	159	11.1	3	0.2	2,352	392	16.7	378	16.1	14	0.6
*Klebsiella pneumoniae*	437	159	36.4	49	11.2	110	25.1	260	36	13.8	16	6.2	20	7.7	697	195	28.0	65	9.3	130	18.7
*Proteus mirabilis*	152	39	25.7	39	25.7	0	NA	123	26	21.1	26	21.1	0	NA	275	65	23.6	65	23.6	0	NA
Total target species	1,509	428	28.4	309	20.3	121	8.0	1,815	224	12.3	201	11.1	23	1.3	3,324	652	19.6	508	15.3	144	4.3

ESCR: non-susceptible to extended-spectrum cephalosporins; ESCR-carbaS: non-susceptible to extended-spectrum cephalosporins but susceptible to carbapenems; ESCR-carbaR: isolates non-susceptible to extended-spectrum cephalosporins and non-susceptible to carbapenems; NA: not applicable.

Overall, 508 isolates were confirmed to be non-susceptible to ESC but susceptible to carbapenems (ESCR-carbaS phenotype), and 144 isolates to be non-susceptible to both ESC and carbapenems (ESCR-carbaR). Isolates susceptible to ESC and non-susceptible to carbapenems were not detected.

The ESCR-carbaS phenotypes were contributed by all three species, with higher proportions among *P. mirabilis* (23.6%) and *E. coli* (16.1%) than among *K. pneumoniae* (9.3%), and with a higher proportion among isolates from inpatients (20.3%) but a remarkable proportion (11.1%) also among those from outpatients (Table 2).

The ESCR-carbaR phenotypes were mostly contributed by *K. pneumoniae*, with higher proportion among isolates from inpatients (25.1% of *K. pneumoniae*) but also a notable proportion among those from outpatients (7.7% of *K. pneumoniae*) (Table 2).

Considering the nature of resistant isolates, the majority of those from outpatients (n=224) were from urine (206/224, 91.9%), while those from inpatients (n=428) were from different specimens (urine: 239/428, 55.8%; blood: 55/428, 12.8%; respiratory tract samples: 54/428, 12.6%).

### Beta-lactamase phenotypes and genotypes of the ESCR and carbapenem-resistant isolates

**Figure 2:**
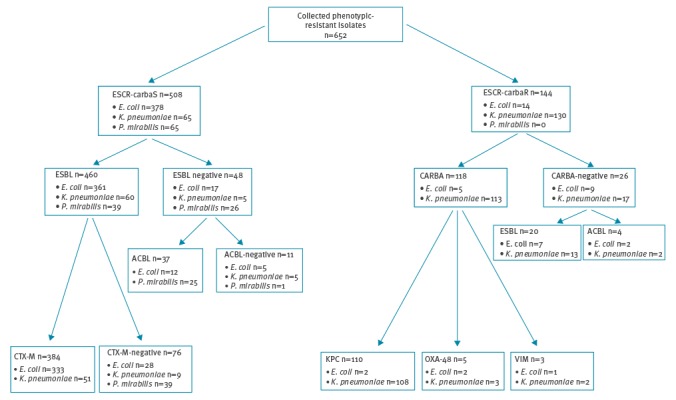
Distribution of *Escherichia coli*, *Klebsiella pneumoniae* and *Proteus mirabilis* isolates according to resistance phenotypes and genotypes, nationwide surveillance survey, Italy, October 2013 (n=652 isolates)

ACBL: isolates producing acquired class C beta-lactamase; CARBA: carbapenemase producing isolates; *E. coli*: *Escherichia coli;* ESBL: isolates producing extended spectrum beta-lactamase; ESCR-carbaS: isolates non-susceptible to extended-spectrum cephalosporins but susceptible to carbapenems; ESCR-carbaR: isolates non-susceptible to extended-spectrum cephalosporins and non-susceptible to carbapenems; *K. pneumoniae*: *Klebsiella pneumoniae*; *P. mirabilis*: *Proteus mirabilis*.

Of the ESCR-carbaS isolates, 460 (90.5%) were ESBL-positive and 384 of them (83.4%) carried a *bla*_CTX-M_ ESBL gene (Figure 2 and Table 3).

**Table 3:** Resistance mechanisms detected in the investigated phenotypic resistant isolates by species included, nationwide surveillance survey, Italy, October 2013 (n=652 isolates)

Phenotypic resistance	Resistance mechanism	*Escherichia coli*		*Klebsiella pneumoniae*		*Proteus mirabilis*		Total	
	**N**	**%**	**n**	**%**	**n**	**%**	**n**	**%**
**Total**	**2,352**	**100**	**697**	**100**	**275**	**100**	**3,324**	**100**
Resistant	392	16.7	195	28.0	65	23.6	652	19.6
**ESCR-carbaS**	**Total**	**378**	**96.4**	**65**	**33.3**	**65**	**100.0**	**508**	**77.9**
ESBL	CTX-M-1	264	69.8	50	76.9	0	NA	314	61.8
	CTX-M-9	61	16.1	0	NA	0	NA	61	12.0
	CTX-M-1 + 9	8	2.1	1	1.5	0	NA	9	1.8
	Other ESBL	28	7.4	9	13.8	39	60.0	76	15.0
ACBL	CMY/LAT/ACT/MIR	12	3.2	0	NA	24	36.9	36	7.1
	DHA	0	NA	0	NA	1	1.5	1	0.2
Other	Other resistance mechanism	5	1.3	5	7.7	1	1.5	11	2.1

**ESCR-carbaR**	**Total**	**14**	**3.6**	**130**	**66.7**	**0**	**0.0**	**144**	**22.1**
CARBA	KPC	2	14.3	108	83.2	0	NA	110	76.4
	VIM	1	7.1	2	1.5	0	NA	3	2.1
	OXA-48	2	14.3	3	2.3	0	NA	5	3.5
ESBL	CTX-M-1	4	28.6	9	6.9	0	NA	13	9.0
	CTX-M-9	3	21.4	0	NA	0	NA	3	2.1
	CTX-M-1 + 9	0	NA	1	0.8	0	NA	1	0.7
ACBL	CMY/LAT/ACT/MIR	2	14.3	0	NA	0	NA	2	1.4
	DHA	0	NA	2	1.5	0	NA	2	1.4
Other	Other resistance mechanism	0	NA	5	3.8	0	NA	5	3.5

**Total**	**Total**	**392**	**100**	**195**	**100**	**65**	**100**	**652**	**100**
CARBA	KPC	2	0.5	108	55.4	0	NA	110	16.9
	VIM	1	0.3	2	1.0	0	NA	3	0.5
	OXA-48	2	0.5	3	1.5	0	NA	5	0.8
ESBL	CTX-M-1	268	68.4	59	30.3	0	NA	327	50.2
	CTX-M-9	64	16.3	0	NA	0	NA	64	9.8
	CTX-M-1 + 9	8	2.0	2	1.0	0	NA	10	1.5
ACBL	CMY/LAT/ACT/MIR	14	3.6	0	NA	24	36.9	38	5.8
	DHA	0	NA	2	1.0	1	1.5	3	0.5
Other	Other resistance mechanism	33	8.4	19	9.7	40	61.5	92	14.1

ACBL: acquired class C beta-lactamase-producing; CARBA: carbapenemase-producing; CTX-M-1: CTX-M group 1 producers; CTX-M-9: CTX-M group 9 producers; CTX-M-1+9: CTX-M group 1 and group 9 producers; ESBL: extended-spectrum beta-lactamase-producing; ESCR-carbaR: non-susceptible to extended-spectrum cephalosporins and non-susceptible to carbapenems; ESCR-carbaS: non-susceptible to extended-spectrum cephalosporins but susceptible to carbapenems; NA: not applicable.

The proportion of *bla*_CTX-M_ genes was 92.2% among ESBL-positive *E. coli* and 84.7% among ESBL-positive *K. pneumoniae*, while such genes were never detected among ESBL-positive isolates of *P. mirabilis* (Figure 2). Among CTX-M-positive isolates, most carried *bla*_CTX-M_ genes of group 1 (81.5%), while a minority carried *bla*_CTX-M_ genes of group 9 (16,0%) and a small proportion carried *bla*_CTX-M_ genes of both CTX-M groups (2.5%) (Table 3). CTX-M-positive isolates were detected from all centers (Figure 1).

Comparison of these data with results from the earlier Italian nationwide survey on producing ESBL-producing *Enterobacteriaceae*, carried out in 2003 [29], revealed a notable increase in the proportion of ESBL-producing isolates (from 6.4% to 14.4%; p < 0.001, considering the three target species). Among outpatients, the proportion of ESBL producers increased from 3.4% to 11.0% (p < 0.001), and the increase was especially large among *E. coli* (from 1.9% to 10.9%; p < 0.001).

The majority of the ESCR-carbaS isolates that were ESBL-negative were positive for an acquired ACBL gene (n = 37, 79%) which, in most cases, belonged to the CMY/LAT/ACT/MIR lineage. Most of the ACBL-positive isolates were *P. mirabilis* (Figure 2, Table 3).

Concerning the ESCR-carbaR isolates, most (81.9%) were positive for carbapenemase genes, including *bla*_KPC_- (93.2%), *bla*_OXA-48_- (4.2%), and *bla*_VIM_-type (2.6%) carbapenemase genes. The majority of carbapenemase-producers were *K. pneumoniae*, while only five *E. coli* produced a carbapenemase (Figure 2). Interestingly, some KPC-positive *K. pneumoniae* (KPC-KP) (6.5%) and most OXA-48-positive isolates also harbored a *bla*_CTX-M_ gene (Table 3). The 26 ESCR-carbaR isolates that tested negative for carbapenemase genes were confirmed to be carbapenemase non-producers by spectrophotometric assay. Most of these isolates carried *bla*_CTX-M_ genes (65.3%) while a minority was positive for an ACBL gene (*bla*_DHA_ or *bla*_CMY/LAT/ACT/MIR_), suggesting that in these cases the carbapenem resistance mechanism was related with production of an ESBL or ACBL in combination with reduced permeability [30].

Comparison of these data with results from the first Italian nationwide survey on carbapenem-resistant *Enterobacteriaceae* (CRE), carried out in 2011 [22], revealed an increase in the proportion of carbapenem-resistant *K. pneumoniae* isolates (from 11.9% to 18.7%) although the difference was not statistically significant (p = 0.5). KPC-KP remained the major contributors (83.1% of carbapenem-resistant *K. pneumoniae* in 2013 *vs*. 87.2% in 2011). Among outpatients, the proportion of KPC-KP increased from 2.2% in 2011 to 4.6% (p < 0.05).

A detailed distribution of the resistance genes in the Italy is reported in Figure 2, and the distribution by species and type of patients is reported in Table 2.

### Antimicrobial susceptibility

All ESBL-producing isolates that were carbapenemase-negative remained susceptible to imipenem and meropenem, while ertapenem susceptibility was 98.1% among *E. coli* and 82.2% among *K. pneumoniae* (Table 4).

**Table 4:** Antimicrobial susceptibility testing results for the investigated isolates, nationwide surveillance survey, Italy, October 2013 (n=652 isolates)

** Species**	**Phenotypic resistance**	**n**	Susceptible (%)

AK	GM	CIP	CAZ	CTX	FEP	IMI	MEM	ERT	AZT	TRIM/SUL	COL^a^	TIG^a^
*Escherichia coli*	ESBL	368	94.8	61.7	9.8	10.6	0.8	14.9	100.0	100.0	98.1	6.3	43.8	NA	NA
	CARBA	5	80.0	60.0	20.0	20.0	0.0	20.0	80.0	60.0	0.0	20.0	40.0	100.0	100.0
	Others	19	100.0	89.5	52.6	0.0	0.0	94.7	100.0	100.0	89.5	5.3	42.1	NA	NA
	Total	392	94.9	63.0	12.0	10.2	0.8	18.9	99.7	99.5	96.4	6.4	43.6	NA	NA
*Klebsiella pneumoniae*	ESBL	73	91.8	39.7	21.9	4.1	6.8	6.8	100.0	100.0	82.2	8.2	26.0	NA	NA
	CARBA	113	16.8	48.7	0.9	0.0	0.0	0.0	3.5	3.5	0.0	0.0	25.7	61.9	94.4
	Others	9	66.7	88.9	44.4	0.0	66.7	77.8	100.0	88.9	55.6	77.8	55.6	NA	NA
	Total	195	47.2	47.2	10.8	1.5	5.6	6.2	44.1	43.6	33.3	6.7	27.2	NA	NA
*Proteus mirabilis*	ESBL	39	92.3	0.0	30.8	43.6	7.7	61.5	NA	100.0	100.0	87.2	25.6	NA	NA
	CARBA	0	NA	NA	NA	NA	NA	NA	NA	NA	NA	NA	NA	NA	NA
	Others	26	96.2	57.7	7.7	0.0	0.0	88.5	NA	100.0	100.0	100.0	7.7	NA	NA
	Total	65	93.8	23.1	21.5	26.2	4.6	72.3	NA	100.0	100.0	92.3	18.5	NA	NA

AK: amikacin; AZT: aztreonam; CARBA: carbapenemase production; CAZ: ceftazidime; CIP: ciprofloxacin; COL: colistin ; CTX: ceftriaxone; ERT: ertapenem; ESBL: extended spectrum beta-lactamase production; FEP: cefepime; GM: gentamicin; IMI: imipenem; MEM: meropenem; NA: not applicable; TIG: tigecycline; TRIM/SUL: trimethoprim/sulfamethoxazole .

^a^ COL and TIG were tested only for carbapenemase producers.

Among the carbapenemase producers, all were non-susceptible to ertapenem, while most carbapenemase-producing *E. coli* were susceptible to imipenem and meropenem (80.0% and 60.0%, respectively). Carbapenemase-producing *K. pneumoniae* were more susceptible to gentamicin than to amikacin (48.7% *vs*. 16.8%). Broth microdilution assays, carried out with the 113 carbapenemase-producing *K. pneumoniae*, revealed that 61.7% and 94.4% were susceptible to colistin and tigecycline, respectively, while all five carbapenemase-producing *E. coli* were susceptible to both drugs (Table 4). These results were overall similar to those reported from the European Survey of Carbapenemase-Producing Enterobacteriaceae (EuSCAPE) survey in 2015 [31], and underscore the remarkable rate of colistin resistance among carbapenemase-producing *K. pneumoniae* circulating in Italy. No significant differences in susceptibility to these two drugs were detected among inpatients and outpatients (data not shown).

### Screening for *mcr-1*-like genes in colistin-resistant isolates

The 43 colistin-resistant carbapenemase-positive *K. pneumoniae* isolates were screened for the presence of *mcr-1*-like genes, encoding transferable colistin resistance [32,33]. All the tested isolates yielded negative results, revealing that colistin resistance was caused by different mechanisms.

## Discussion

This study provides an updated picture of the prevalence, distribution, beta-lactamase profiles and susceptibilities of ESBL-, carbapenemase- and ACBL-producing *E. coli*, *K. pneumoniae* and *P. mirabilis* circulating in Italy.

Compared with the earlier Italian nationwide survey on ESBL-producing *Enterobacteriaceae*, carried out in 2003 [29], the proportion of ESBL-producing isolates (considering the three target species) showed a notable increase, especially among outpatients and among *E. coli*. At the level of resistance mechanisms, this epidemiological evolution was associated with a remarkable increase in the prevalence of CTX-M-type enzymes, which, in 2013, represented by far the most common type of ESBL in *E. coli* and in *K. pneumoniae*. Interestingly, despite their ability to spread, the genes encoding these resistance mechanisms have not disseminated in *P. mirabilis*, where the ESCR phenotype was found to be relatively common (23.6% of isolates) but due to other mechanisms, i. e. ESBLs other than CTX-M-type or ACBLs, mostly of the CMY/LAT/ACT/MIR lineage. A similar epidemiological evolution was observed in other European Countries including Belgium (with 77% vs 46% of CTX-M-positive isolates, among ESBL-producing *E. coli*, in 2008 vs 2006) [34], Spain (with 72% vs 52% of CTX-M- positive isolates among ESBL-producing *E. coli* in 2006 vs 2000) [35].

Concerning carbapenem resistance, a notable increase was observed among *K. pneumoniae* in comparison with data from the first Italian nationwide survey on CRE, carried out in 2011 [22], with KPC-KP remaining the major contributors of CRE endemicity in Italy and other types of carbapenemases remaining uncommon. These findings underscore the ability of carbapenemase-producing *Enterobacteriaceae* (CPE) to rapidly disseminate and establish conditions of high-level endemicity, and the notion that CPE are associated with a heightened epidemiological risk and deserve special attention, as readily acknowledged by the European Centre for Disease Prevention and Control (ECDC) [36]. Indeed, the data from the EARS-NET surveillance system indicate that there is an overall increasing spread of CRE in some European countries [21], and results from the EuSCAPE project have shown that some regions are experiencing a worrisome prevalence of CPE per 10,000 hospital admissions, ranging from four to six for Italy, Greece and Spain [37].

Interestingly, KPC-KP isolates were also found from outpatients, at a rate that was approximately double compared with that found in the 2011 survey (4.6% vs 2.2%) [22]. Since a significant proportion of these outpatients could have been recently hospitalised or otherwise have had contact with healthcare services, this figure may not reflect accurately the prevalence of KPC-KP in the community, in Italy. However, this finding points to an increasing dissemination of CPE also outside the hospital setting, likely reflecting the ability of KPC-KP to establish persistent intestinal colonisation even after hospital discharge [38,39]. It might herald a possible dissemination in the community like the one previously witnessed with CTX-M-producing *E. coli* [40]. Indeed, recent reports from the US and Spain have described the emergence of CPE infections also in the community, underscoring this possibility [41,42]. Such a scenario should be avoided, and suitable infection control measures should be enforced to address this phenomenon in settings of high CRE endemicity like Italy and some other European countries [37].

In this grim scenario, the good news was that (i) overall carbapenem susceptibility was retained by *P. mirabilis*, with no carbapenemase-positive isolates detected in this species despite the high endemicity of CPE; and that (ii) no *mcr-1*-like transferable colistin resistance genes were detected among the colistin-resistant CPE isolates, suggesting that this worrisome resistance mechanism remains uncommon among colistin-resistant CPE circulating in Italy.

Updated molecular surveillance data on resistance genes to beta-lactams are of increasing importance due to the introduction on the market of new antibiotics for resistant Gram-negatives that are based on new beta-lactamase inhibitors (e. g. avibactam), which will be useful for treatment of infections caused by ESBL and carbapenemase producers [43]. The new inhibitors, however, are not active against all beta-lactamases e. g. avibactam is able to inhibit class A but not class B beta-lactamases. Thus the possibility of using these new drugs will depend on the specific mechanism of resistance, and rapid testing for these resistance mechanisms will become an essential step in antibiotic stewardship programs. In this perspective, this survey underscores the major role of beta-lactamase genes as resistance determinants in *Enterobacteriaceae* in the Italian epidemiological setting, and provides relevant information for the selection of the most suitable diagnostic strategies based on molecular detection of beta-lactam resistance mechanisms. A limitation of this study is that, due to the study design, laboratory-based surveillance, information about risk factors for developing antibiotic resistance was not available. Future studies are warranted to investigate these aspects, which are relevant to understand the reasons for the epidemiological differences observed in different European countries and to contextualise infection control policies.

## Notes

**AMCLI-CoSA Survey participants:** Milan, Niguarda Ca’ Granda Hospital – C. Vismara; Lecco, A. Manzoni Hospital – B. Pini; Novara, Maggiore Hospital – S. Andreoni; San Remo, Azienda Sanitaria Imperiese Hospital – P. A. Dusi; Bolzano, Azienda Sanitaria dell’Alto Adige Hospital – R. Aschbacher; Udine, Azienda Ospedaliera Santa Maria della Misericordia – C. Scarparo; Modena, Baggiovara Hospital – M. Sarti; Modena, Modena University Hospital –C. Venturelli; Florence, Careggi University Hospital – P. Pecile; Ancona, ‘Torrette’ University Hospital – E. Manso; Rome, Policlinico Gemelli – T. Spanu; San Giovanni Rotondo, IRCCS Casa Sollievo della Sofferenza Hospital – M. Labonia; Avellino, San Giuseppe Moscati Hospital – G. Buonopane; Cosenza, Annunziata Hospital – C. Giraldi.
