# Erratum for Euro Surveill. 2017;22(31)

**DOI:** 10.2807/1560-7917.ES.2017.22.34.30601

**Published:** 2017-08-24

**Authors:** Eurosurveillance editorial team

**Affiliations:** 1 European Centre for Disease Prevention and Control (ECDC), Stockholm, Sweden

In the article ‘Evolving beta-lactamase epidemiology in Enterobacteriaceae from Italian nationwide surveillance, October 2013: KPC-carbapenemase spreading among outpatients’, published on 3 August 2017, the cells in the topmost rows of the Tables 2, 3 and 4 were erroneously shifted to the left. The tables were corrected and replaced on 21 August 2017.

